# In situ recording of Mars soundscape

**DOI:** 10.1038/s41586-022-04679-0

**Published:** 2022-04-01

**Authors:** S. Maurice, B. Chide, N. Murdoch, R. D. Lorenz, D. Mimoun, R. C. Wiens, A. Stott, X. Jacob, T. Bertrand, F. Montmessin, N. L. Lanza, C. Alvarez-Llamas, S. M. Angel, M. Aung, J. Balaram, O. Beyssac, A. Cousin, G. Delory, O. Forni, T. Fouchet, O. Gasnault, H. Grip, M. Hecht, J. Hoffman, J. Laserna, J. Lasue, J. Maki, J. McClean, P.-Y. Meslin, S. Le Mouélic, A. Munguira, C. E. Newman, J. A. Rodríguez Manfredi, J. Moros, A. Ollila, P. Pilleri, S. Schröder, M. de la Torre Juárez, T. Tzanetos, K. M. Stack, K. Farley, K. Williford, the SuperCam team, R. C. Wiens, T. Acosta-Maeda, R. B. Anderson, D. M. Applin, G. Arana, M. Bassas-Portus, R. Beal, P. Beck, K. Benzerara, S. Bernard, P. Bernardi, T. Bosak, B. Bousquet, A. Brown, A. Cadu, P. Caïs, K. Castro, E. Clavé, S. M. Clegg, E. Cloutis, S. Connell, A. Debus, E. Dehouck, D. Delapp, C. Donny, A. Dorresoundiram, G. Dromart, B. Dubois, C. Fabre, A. Fau, W. Fischer, R. Francis, J. Frydenvang, T. Gabriel, E. Gibbons, I. Gontijo, J. R. Johnson, H. Kalucha, E. Kelly, E. W. Knutsen, G. Lacombe, S. Le Mouélic, C. Legett, R. Leveille, E. Lewin, G. Lopez-Reyes, E. Lorigny, J. M. Madariaga, M. Madsen, S. Madsen, L. Mandon, N. Mangold, M. Mann, J.-A. Manrique, J. Martinez-Frias, L. E. Mayhew, T. McConnochie, S. M. McLennan, N. Melikechi, F. Meunier, G. Montagnac, V. Mousset, T. Nelson, R. T. Newell, Y. Parot, C. Pilorget, P. Pinet, G. Pont, F. Poulet, C. Quantin-Nataf, B. Quertier, W. Rapin, A. Reyes-Newell, S. Robinson, L. Rochas, C. Royer, F. Rull, V. Sautter, S. Sharma, V. Shridar, A. Sournac, M. Toplis, I. Torre-Fdez, N. Turenne, A. Udry, M. Veneranda, D. Venhaus, D. Vogt, P. Willis

**Affiliations:** 1 Institut de Recherche en Astrophysique et Planétologie, Université de Toulouse 3 Paul Sabatier, CNRS, CNES, Toulouse, France; 2 Space and Planetary Exploration Team, Los Alamos National Laboratory, Los Alamos, NM USA; 3 Institut Supérieur de l’Aéronautique et de l’Espace (ISAE-SUPAERO), Université de Toulouse, Toulouse, France; 4 Space Exploration Sector, Johns Hopkins Applied Physics Laboratory, Laurel, MD USA; 5 Present Address: Department of Earth, Atmospheric, and Planetary Sciences, Purdue University, West Lafayette, IN USA; 6 Institut de Mécanique des Fluides, Université de Toulouse 3 Paul Sabatier, INP, CNRS, Toulouse, France; 7 Laboratoire d’Etudes Spatiales et d’Instrumentation en Astrophysique, Observatoire de Paris, CNRS, Sorbonne Université, Université Paris Diderot, Meudon, France; 8 Laboratoire Atmosphères, Milieux, Observations Spatiales, CNRS, Université Saint-Quentin-en-Yvelines, Sorbonne Université, Guyancourt, France; 9 Universidad de Málaga, Málaga, Spain; 10 Department of Chemistry and Biochemistry, University of South Carolina, Columbia, SC USA; 11 Jet Propulsion Laboratory, California Institute of Technology, Pasadena, CA USA; 12 Institut de Minéralogie, de Physique des Matériaux et de Cosmochimie, CNRS, Sorbonne Université, MNHN, Paris, France; 13 Heliospace Corporation, Berkeley, CA USA; 14 Haystack Observatory, Massachusetts Institute of Technology, Westford, MA USA; 15 Department of Aeronautics and Astronautics, Massachusetts Institute of Technology, Cambridge, MA USA; 16 Laboratoire de Planétologie et Géosciences, CNRS, Nantes Université, Université Angers, Nantes, France; 17 Escuela de Ingeniería de Bilbao, Universidad del País Vasco UPV/EHU, Bilbao, Spain; 18 Aeolis Corporation, Sierra Madre, CA USA; 19 Centro de Astrobiología (INTA-CSIC), Madrid, Spain; 20 Deutsches Zentrum für Luft- und Raumfahrt (DLR), Institute of Optical Sensor Systems, Berlin, Germany; 21 Blue Marble Space Institute of Science, Seattle, WA USA; 22 University of Hawai‘i at Mānoa, Mānoa, HI USA; 23 U.S. Geological Survey, Flagstaff, AZ USA; 24 University of Winnipeg, Winnipeg, Canada; 25 University of the Basque Country UPV/EHU, Leioa, Bilbao Spain; 26 Institut de Planétologie et Astrophysique de Grenoble, CNRS, Université Grenoble Alpes, Grenoble, France; 27 Department of Earth, Atmospheric and Planetary Sciences, Massachusetts Institute of Technology, Cambridge, MA USA; 28 Centre Lasers Intenses et Applications, CNRS, CEA, Université de Bordeaux, Bordeaux, France; 29 Plancius Research, Severna Park, MD USA; 30 Laboratoire d’Astrophysique de Bordeaux, CNRS, Université de Bordeaux, Bordeaux, France; 31 Centre National d’Études Spatiales, Toulouse, France; 32 Université de Lyon, UCBL, ENSL, UJM, CNRS, LGL-TPE, Villeurbanne, France; 33 Groupe d’Instrumentation Scientifique, Observatoire Midi-Pyrénées, Toulouse, France; 34 GeoRessources, CNRS, Université de Lorraine, Nancy, France; 35 California Institute of Technology, Pasadena, CA USA; 36 University of Copenhagen, Copenhagen, Denmark; 37 McGill University, Montreal, Canada; 38 University of Valladolid, Valladolid, Spain; 39 Agencia Estatal Consejo Superior de Investigaciones Científicas, Madrid, Spain; 40 Department of Geological Sciences, University of Colorado Boulder, Boulder, CO USA; 41 University of Maryland, College Park, MD USA; 42 State University of New York, Stony Brook, NY USA; 43 Department of Physics and Applied Physics, Kennedy College of Sciences, University of Massachusetts Lowell, Lowell, MA USA; 44 Institut d’Astrophysique Spatiale, CNRS, Université Paris-Saclay, Orsay, France; 45 Institut Universitaire de France, Paris, France; 46 University of Nevada, Las Vegas, Las Vegas, NV USA

**Keywords:** Atmospheric dynamics, Atmospheric dynamics, Characterization and analytical techniques

## Abstract

Before the Perseverance rover landing, the acoustic environment of Mars was unknown. Models predicted that: (1) atmospheric turbulence changes at centimetre scales or smaller at the point where molecular viscosity converts kinetic energy into heat^1^, (2) the speed of sound varies at the surface with frequency^2,3^ and (3) high-frequency waves are strongly attenuated with distance in CO_2_ (refs. ^2–4^). However, theoretical models were uncertain because of a lack of experimental data at low pressure and the difficulty to characterize turbulence or attenuation in a closed environment. Here, using Perseverance microphone recordings, we present the first characterization of the acoustic environment on Mars and pressure fluctuations in the audible range and beyond, from 20 Hz to 50 kHz. We find that atmospheric sounds extend measurements of pressure variations down to 1,000 times smaller scales than ever observed before, showing a dissipative regime extending over five orders of magnitude in energy. Using point sources of sound (Ingenuity rotorcraft, laser-induced sparks), we highlight two distinct values for the speed of sound that are about 10 m s^−1^ apart below and above 240 Hz, a unique characteristic of low-pressure CO_2_-dominated atmosphere. We also provide the acoustic attenuation with distance above 2 kHz, allowing us to explain the large contribution of the CO_2_ vibrational relaxation in the audible range. These results establish a ground truth for the modelling of acoustic processes, which is critical for studies in atmospheres such as those of Mars and Venus.

## Abstract (web summary)

Using data gathered from the microphones of the Perseverance rover, the first characterization of the acoustic environment on Mars is presented, showing two distinct values for the speed of sound in CO_2_-dominated atmosphere.

## Main

Before the landing of Perseverance (18 February 2021), no pressure fluctuations had ever been monitored on Mars at a frequency >20 Hz, namely, in the acoustic domain. The recording of sounds offers the unique opportunity to study the atmosphere as the main natural source of sound and as the propagation medium for acoustic waves. From the knowledge of Mars atmospheric pressure (about 0.6 kPa) and the physical properties of CO_2_, one can predict (see Methods) that: the acoustic impedance results in approximately 20 dB weaker sounds on Mars than on Earth if produced by the same source, the speed of sound should be around 240 m s^−1^ near the surface and acoustic waves are heavily damped in CO_2_ at these atmospheric pressures and temperatures. A few studies^2,3^ proposed very detailed models of acoustic propagation on Mars but with large discrepancies between their results because of a lack of experimental data at low pressure and appropriate temperatures, and the difficulty of characterizing attenuation in a closed environment. Acoustic data are also sensitive to wind speed and direction and, to a lesser extent, other environmental parameters^5,6^. As such, owing to the high sampling frequency of microphones (up to 100 kHz), the acoustic data allow us to explore the atmospheric behaviour on a microscale that has never been accessible before on Mars.

The SuperCam instrument suite^7,8^ on Perseverance carries an electret microphone, similar to that carried by the Mars Polar Lander^9^, lost during atmospheric entry, and the Phoenix spacecraft^10^, on which technical issues prevented the device from being operated. SuperCam’s microphone is able to record air pressure fluctuations from 20 Hz to 12.5 kHz or 50 kHz, at sampling rates of 25 kHz or 100 kHz, respectively. After landing (Martian solar day ‘Sol’ 0; one Sol = 88,775 s), the microphone was turned on for the first time on Sol 1 while the mast was still stowed. Since deployment on Sol 2, the microphone is approximately 2.1 m above the ground; it has performed nominally up to the time of writing. SuperCam also consists of a laser-induced breakdown spectroscopy (LIBS) capability to analyse the chemistry of Mars at stand-off distances from 1.5 to 7 m (refs. ^7,8^). When the laser pulse interacts with the target, a luminous plasma emits characteristic optical emission lines of the elements present in the target^11^. Plasma expansion generates a shock wave that decouples from the plasma within the first microsecond after laser interaction^12^ and results in a clearly detectable acoustic signal^13,14^. Moreover, Perseverance carries a second microphone as part of the Entry, Descent, and Landing Camera (EDLCAM^15^), which has a frequency response from 20 Hz to 20 kHz at a sampling rate of 48 kHz. The EDL microphone is mounted on the port side of the rover, 1 m above the ground. It was activated on Sol 2.

**Fig. 1:**
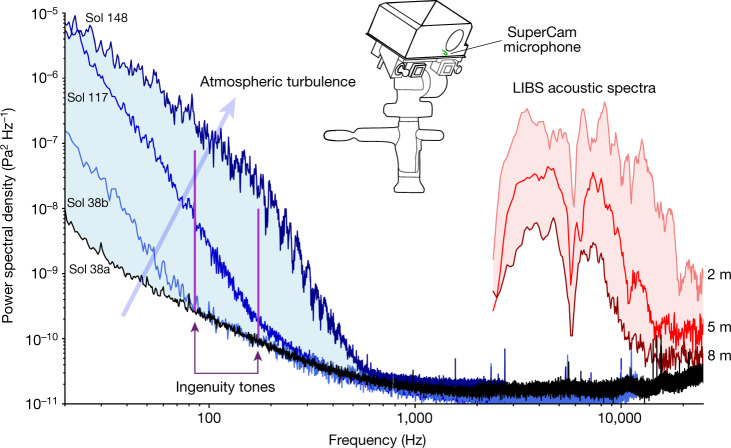
Variety of sounds recorded by SuperCam. Atmospheric spectra spread over the light blue area; turbulence increases in the direction of the arrow. LIBS acoustic spectra spread over the light red area. Ingenuity tones are recorded at 84 Hz and 168 Hz (purple). The black spectrum is the quietest recording so far below 1 kHz. SuperCam’s microphone is located on the rover mast (green).

Figure 1 provides an overview of sounds acquired by SuperCam’s microphone (see Methods). Sol 38a is the quietest recording in our dataset. Later on that same day (Sol 38b), the power spectral density (PSD) increases above the quiet state at frequencies below 100 Hz. On Sol 117, we associate this increase of power to an increase in the turbulent activity, which extends up to 300 Hz; this is the situation we observe most often. The recording of Sol 148 is the most active one shown, with the same shape starting towards higher frequencies but with a slope break near 200 Hz; turbulence is detected up to 600 Hz. All non-saturated atmospheric recordings from Sol 0 to Sol 216 fit between the boundaries given by the Sol 38a and Sol 148 spectra. The laser-excited plasma generates a short, roughly 300-μs acoustic pulse (see Methods), with 95% of its energy between 3 and 15 kHz. Various spectral notches are caused by acoustic interferences owing to echoes from the base of the microphone itself (6 kHz and 12 kHz) or from nearby rocks. The total intensity varies as a function of target distance, as shown for recordings at 2 m, 5 m and 8 m. During laser-induced spark recording sessions, the atmospheric signal below 1 kHz is masked by electromagnetic interference^8^. The Ingenuity rotorcraft tones (see Methods) are also shown.

## Atmospheric turbulence

The Martian planetary boundary layer (PBL) is the part of the atmosphere in contact with the surface^16^, extending to several km. It is prone to convective turbulence and vertical mixing during daytime, owing to the thin atmosphere and low surface thermal inertia that induce strong and unstable near-surface temperature gradients^17–19^. This turbulence translates into high-frequency variations in atmospheric pressure, wind speed and temperature that can be measured by in situ instruments. Conversely, during night-time, the strong radiative cooling of the atmosphere induces highly stable conditions, which efficiently inhibit most convection and turbulence^16^. Analysing the PBL at the surface is therefore important to understand how the Martian atmosphere transports and mixes heat, momentum, aerosols and chemical species^20^. The Mars Environmental Dynamics Analyzer (MEDA^21^) instrument on Perseverance and the meteorological suites of previously landed missions^20,22^ typically measure pressure, temperature and wind fluctuations with sampling frequencies of 0.1 Hz to 10 Hz. These instruments study the turbulence variability^23,24^ and the Martian turbulent energy cascade^1,17,25^.

Specifically, we report here the observation of the dissipative turbulence regime in the PBL, in which the InSight mission could see a hint of a regime change at the limits of the instrument capability^1^. This regime, in which molecular viscosity dissipates the turbulent kinetic energy into heat, is now fully characterized by a rapid decrease of the power spectrum with increasing frequency (Fig. 1, 2b) over roughly five orders of magnitude. The scale at which the viscous dissipation becomes notable is characterized by the Kolmogorov length scale^26^, *η* = (*ν*^3^/*ε*)^0.25^, in which *ν* is the kinematic viscosity and *ε* is the turbulence energy dissipation rate per unit mass, typically around 0.001 m^2^ s^−1^ and 0.005 m^2^ s^−1^ on Mars, respectively^17^. Thus *η* is about 0.02 m and the timescale of these small eddies, *t*_*η*_ = (*ν*/*ε*)^0.5^, is about 0.45 s. Hence the dissipation regime should be observable at frequencies above 2 Hz on Mars, at centimetre or smaller scales only (on Earth, this transition occurs at millimetre scales or smaller^17^). This theoretical prediction is confirmed by the acoustic data; the threshold moves with frequency, depending on the dissipation rate^25,27^. The balance between energy production and molecular dissipation controls the total amount of turbulent kinetic energy in the boundary layer and, as such, the dissipation mechanism is intrinsically linked to the PBL dynamics; a larger dissipation leads to a faster turbulence decay, in turn suppressing small-scale wind gustiness, and vice versa.

**Fig. 2:**
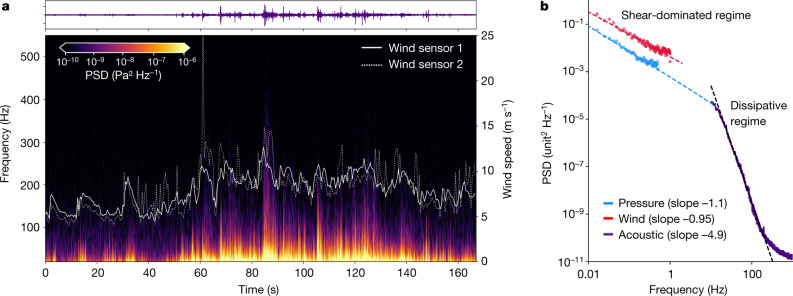
Sound recordings and correlation with atmospheric data. Recording of Sol 38b. **a**, On top, the *y* axis of the time series ranges from −0.2 to 0.2 Pa. The spectrogram (bottom) shows bursts that extend to 300 Hz. Overlaid, with the *y* axis on the right, are wind speeds from MEDA booms. **b**, The PSD calculated for SuperCam’s microphone (in Pa^2^ Hz^−1^ for 167 s) and for MEDA pressure (in Pa^2^ Hz^−1^ for 51 min around the microphone acquisition time) and MEDA wind data (in (m s^−1^)^2^ Hz^−1^). The wind PSD is artificially offset by 10^−2^ in the *y* axis.

The microphone records rapid deviations from ambient pressure (>20 Hz) that are correlated to variations in the wind flow, as shown by Fig. 2a, in which a spectrogram of Sol 38b microphone data (see Methods) is overlaid with the wind speed as measured by the MEDA (see Methods). As expected^6,13^, there is a clear correlation between the intensity of acoustic data and the wind speed. This can be owing to the flow-induced turbulence from the rover/mast itself but also to the direct sensing of the incoming flow fluctuations, seen to be the dominant factor for outdoor microphones in other studies^6^. Moreover, the daytime local turbulence is known to increase for larger ambient wind speeds^24^. The high microphone sampling rate provides an opportunity to observe very intense but short wind gusts, on a timescale of 10 s. In Fig. 2b, the same acoustic data are plotted in the frequency domain and combined with low-frequency measurements of pressure and wind from the MEDA, for a 51-min time period of continuous data around the microphone acquisition. The large difference in slope between the MEDA and microphone data is indicative of regime change. The transition from the probable shear-dominated regime^28^ to the dissipation regime occurs in this case between 1 and 20 Hz.

## Speed of sound on Mars

In a cold CO_2_ atmosphere, the speed of sound is expected to be lower than on Earth. Furthermore, owing to the low pressure and the physical properties of CO_2_, we also expect a dispersion of this speed with frequency^2,3^. On Earth, the adiabatic ratio *γ* is constant up to a few MHz at ambient pressure^29^ and sound speed does not vary with frequency near the surface. At low pressure on Mars, still within the framework of small Knudsen numbers^30^ (10^−6^ at 100 Hz to 2.10^−4^ at 20 kHz), the continuum theory still holds, but energy exchanges at molecular scales are modified. Part of the energy associated with the translational motions of molecules, which constitute the acoustic waves, is spent on the excitation of inner degrees of freedom (vibrational modes and rotational motions). The relaxation of the rotational motion is almost instantaneous, whereas relaxation of the vibrational modes occurs over a much longer timescale, a property of small and rigid polyatomic molecules such as CO_2_. If the frequency *f* is smaller than *f*_R_ = 1/*τ*_R_, in which *τ*_R_ is the relaxation time, all modes are equally excited and then relaxed. The seven degrees of freedom that result from three translational modes, two rotational modes and one doubly-degenerate vibrational mode (*ν*_2_, bending) lead to an adiabatic index *γ*_0_ = 9/7 = 1.2857. Conversely, if *f* > *f*_R_, there is no time to relax the vibrational mode; in that case, there are only five active degrees of freedom and *γ*_∞_ = 7/5 = 1.4. In CO_2_ at Earth-ambient pressure, *f*_R_ is about 40 kHz (ref. ^31^). This frequency depends on the rate at which molecules can collide, hence *f*_R_ is proportional to the pressure. As a result, at 0.6 kPa, the relaxation frequency is about 240 Hz on Mars.

**Fig. 3:**
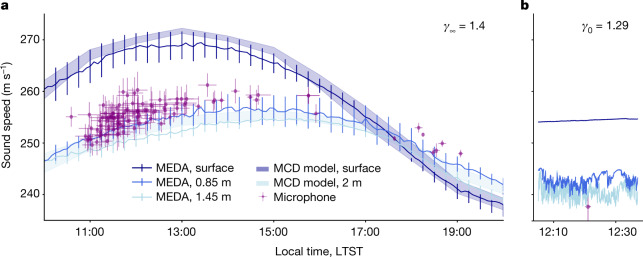
Sound speed variations. **a**, Sound speeds as a function of local time from LIBS time-of-flight data in purple. Other sound speeds are calculated at the three heights from the MEDA temperatures and at the surface and at 2-m altitude from MCD simulations; for these conversions, the adiabatic index above *f*_R_ is used. Error bars for microphone data: standard deviation of the sound speeds during each laser burst (vertical); total duration of the burst (horizontal). Error bars for the MEDA data: standard deviation of 1-h bins between Sols 37 and 216. **b**, Sound speeds are calculated at three heights from the MEDA temperatures during Ingenuity’s fourth flight; the adiabatic index below *f*_R_ is used. The sound speed estimated from the Ingenuity Doppler effect is in purple. Error bars: 95% confidence interval of the Doppler shift fit.

The recording of pulsed waves generated in LIBS mode provides a unique opportunity to measure directly and repetitively the local speed of sound for acoustic waves above 2 kHz, that is, for *f* > *f*_R_ (see Methods). From the daytime measurements, sound speeds between 246 m s^−1^ and 257 m s^−1^ are obtained (Fig. 3a), with maximum values between 11:00 to 14:00 Local True Solar Time (LTST) and minimum values around 18:00. The 1σ-dispersion of the sound speed during the approximately 20 min of a target analysis with LIBS is at its maximum at noon (1.5%) and is reduced to 0.5% at 18:00, which highlights the vanishing of the atmospheric turbulence at dusk. These measurements are compared with temperature-derived speeds of sound obtained from: (1) the MEDA temperature datasets at the surface, at the heights of 0.85 m and 1.45 m, and (2) the temperature at the surface and at a height of 2 m given by the Mars Climate Database (MCD^32^) (see Methods), using *γ*_∞_ = 1.4 (because *f* > *f*_R_). The agreement between the MEDA and MCD predictions is excellent. SuperCam sound speeds are comparable with temperature-derived values at the height of the MEDA’s 0.85-m temperature sensor or higher. This is consistent with the fact that the speed is integrated between a height of 2.1 m and the surface, possibly biased towards the surface when the temperature gradient is larger.

Ingenuity’s blade passage frequency (BPF)^33^ is close to a harmonic source centred around 84 Hz, and — in that case — for *f* < *f*_R_ (see Methods). This signal recorded by SuperCam’s microphone is modulated by the variations of the distance range between the microphone and the helicopter. An emitted frequency at 84.43 Hz and a speed of sound *c* = 237.7 ± 3 m s^−1^ are estimated on the basis of a fit of the Doppler effect for Ingenuity’s fourth flight (see Methods). Accounting for the presence of a wind of about 2.5 m s^−1^ along the microphone-to-helicopter line of sight towards the helicopter (MEDA data), the true sound speed is about 240 m s^−1^ at this frequency. At the time of the flight, the atmospheric temperatures ranged between 232 K and 240 K at a height of 1.45 m. Using *γ*_0_ = 1.2857 (the BPF is below *f*_R_), the temperature-derived speed of sound ranges from 238.8 m s^−1^ to 242.9 m s^−1^, which is consistent with the speed directly derived from Ingenuity’s flight plus wind (Fig. 3b). As a summary, SuperCam’s microphone highlights a sound speed dispersion of about 10 m s^−1^ in the audible range at the surface of Mars.

## Sound attenuation

The most remarkable property of sound propagation on Mars is the magnitude of the attenuation at all frequencies, especially above 1 kHz. The decrease of the LIBS acoustic signal with distance is an opportunity to verify the theory in situ and to test two different attenuation models^3,4^ that suffer from a lack of field data under Mars conditions.

**Fig. 4:**
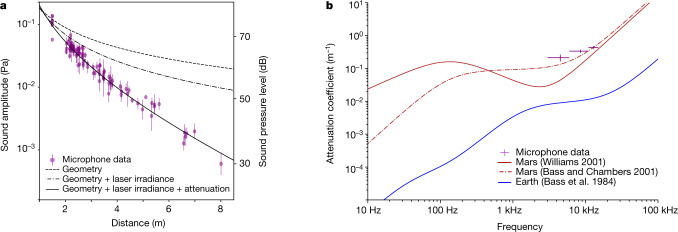
Sound attenuation with distance. **a**, Sound amplitude as a function of target distance *r* from LIBS acoustic data between 6 kHz and 11 kHz. The second vertical axis on the right is for sound pressure level in dB. Signal intensities are in dB relative to 20 μPa. Error bars: standard deviation of the acoustic amplitudes during each laser burst. **b**, Comparison of the attenuation models for Mars^3,4^ (computed at 240 K and 740 Pa) and Earth^34^ (293 K and 30% relative humidity). The experimental points correspond to this study. Error bars: 95% confidence interval of the fit performed in Fig. 4a (vertical) and width of each frequency range (horizontal).

As the spherical LIBS acoustic wave propagates, sound pressure decreases as 1/*r*, in which *r* is the distance between the target and the microphone. This decrease is scaled by a factor *r*^−0.698^ to account for the variation of laser irradiance^8^, multiplied by e^−*αr*^, in which *α* = *α*(*f* ) is the atmospheric attenuation coefficient as a function of frequency. The frequency spectrum of the LIBS acoustic signal is divided into three bands, which account for the three main lobes observed in Fig. 1: from 3 kHz to 6 kHz, from 6 kHz to 11 kHz and from 11 kHz to 15 kHz. The evolution of the sound amplitude with distance for the second frequency band is shown in Fig. 4a. Over the three bands, we find *α* = 0.21 ± 0.04 m^−1^ (95% confidence interval of the fit), *α* = 0.34  ± 0.05 m^−1^ and *α* = 0.43 ± 0.05 m^−1^ respectively. As expected, high-pitched sounds are strongly attenuated. Compared with a signal emitted at 1 m, attenuation of an 8-kHz wave ranges from −9 dB at 2 m to −40 dB at 8 m. At 5 m, the atmospheric absorption takes precedence over the geometrical attenuation. On Earth, for which *α* = 0.01 m^−1^ for the same frequency^34^, the attenuation ranges from −6 dB at 2 m to −20 dB at 8 m, and is almost exclusively resulting from the wavefront spreading. To reach an attenuation of −40 dB on Earth, the source would need to be at 65 m.

Such attenuation coefficients are compared with theoretical^3^ and semi-empirical^4^ attenuation models in Fig. 4b. In situ data tend towards the behaviour described by Bass and Chambers^3^, with a plateau at frequencies <6 kHz and then an increase for higher frequencies. Conversely, the data do not show an attenuation gap as suggested by the model of Williams^4^. This result confirms the large contribution of CO_2_ vibrational relaxation in this frequency range, the same process that explains the two values for the speed of sound (above). However, the attenuation coefficient for the 2–6-kHz band is still higher than that predicted by Bass and Chambers^3^. It may highlight a different relaxation strength than the one forecasted by the model (see Methods). However, these measurements do not reach frequencies low enough to constrain the large discrepancies observed between models below 1 kHz.

## Mars soundscape

Sound is a new, rich source of information on Mars. Thanks to sensors measuring only a few millimetres in diameter, turbulence-induced noise and artificial sources have been recorded. Acoustic waves are governed by the macroscopic thermodynamic properties of fluids (molar mass, heat capacity and temperature, or — alternatively — compressibility and density). However, given the small displacements and timescales that come into play, we confirm that energy exchanges at the molecular scale also need to be considered to accurately model the sound propagation parameter variations (speed, attenuation) with frequency. More sound speed measurements at different local times and seasons will allow the study of atmospheric fluctuations at a scale of a few metres on Mars^35–37^. The first in situ retrieval of the acoustic attenuation coefficient already provides new constraints on theoretical models, which are key parameters for geophysical studies in CO_2_-dominated atmospheres^38,39^. Wind and turbulence, driven by heat fluxes, are natural sources of pressure fluctuations on Mars. We show that acoustic data yield new insights into the boundary layer turbulence with 10 to 1,000 times higher temporal resolution than before, highlighting for the first time the dissipative regime and a transition to this regime above a few Hz. Characterizing this regime in more detail, and the associated transition, is necessary to settle the assumptions used in the numerical modelling of the PBL (including large-eddy simulations), telling us what the fraction of missing energy is in the unresolved scales of the models^40,41^. In the future, this will lead to a measurement of the dissipation rate, related to the diffusion of heat in the atmosphere, which is not well known for Mars at present^17,42^. Finally, beyond the rumble of the wind, the acoustic signatures of our robotic presence on Mars are rich in information on the health of the rover subsystems.

## Methods

This section starts with a reminder on acoustics adapted to Mars. It provides details of the different datasets: acoustic data from the microphones of SuperCam and EDL; artificial sounds from the Mars Oxygen In-Situ Resource Utilization Experiment (MOXIE) and Ingenuity, in addition to the recording of LIBS shock waves and rover noises; wind speed, temperature and pressure data from the MEDA and temperatures extracted from the MCD. Processing methods are presented: computation of PSDs; analyses of LIBS shock wave time series; extraction of the Doppler effect from Ingenuity. Finally, supporting explanations on the attenuation with distance are given.

### Acoustics reminder

We justify the three main assertions in the introduction to the text. First, the acoustic impedance describes the strength of a medium to sustain acoustic waves. It is given by *Z* = *ρc* in the far field of a source, in which *ρ* is the density and *c* is the speed of sound. Typically, with *ρ* = 0.02 kg m^−3^ and *c* = 238 m s^−1^ (see below), we obtain *Z* = 4.76 kg m^−2^ s^−1^ at the surface of Mars, whereas *ρ* = 1.217 kg m^−3^ and *c* = 340 m s^−1^ yield *Z* = 413 kg m^−2^ s^−1^ on Earth. This difference of two orders of magnitude translates into signals on Mars being roughly 20 dB weaker than on Earth when produced by the same source. Second, at Mars pressure, the approximately 95% CO_2_ atmosphere can be efficiently modelled as an ideal gas. In the microphone frequency range, and given the small amplitude of the acoustic pressure, sound waves are considered as adiabatic disturbances. It follows that the temperature-derived speed of sound is given by *c*^2^ = *γRT*/*M*, with *R* the molar gas constant (8.314 J mol^−1^ K^−1^), *M* the molar mass of the atmosphere (43.34 g mol^−1^), *T* the temperature in kelvins and *γ* the adiabatic index. Using *γ* = 9/7, the standard value for CO_2_ — this value is discussed in the main text — we find *c* = 238 m s^−1^ at 230 K. Third, in a rarefied atmosphere, absorption is intrinsically larger as classical (thermal and viscous) and rotational absorption are inversely proportional to the pressure^3^. Moreover, at low frequencies, vibrational absorption dominates over the classical types of absorption and rotation. It turns out that the vibrational specific heat of CO_2_ is 20 times greater than for N_2_ (ref. ^3^) for a comparison with Earth. Hence the doubly degenerate mode of CO_2_ vibration attenuates sounds at low frequencies, whereas the viscosity strongly attenuates frequencies higher than a few kHz. Both effects, in terms of attenuation coefficient per metre, are one or two orders of magnitude stronger than on Earth at the same frequency.

### Acoustic dataset

The SuperCam microphone dataset used in this study extends from Sol 1 to Sol 216, when the first solar conjunction of the mission occurred. At this date, a total of 4 h and 40 min of Martian sounds have been recorded, including atmospheric turbulence (46% of the total duration), the accompanying pressure waves of LIBS sparks (12%) and mechanical noises (for example, MOXIE^43^, Ingenuity^33^, mast rotation of Perseverance, Mastcam-Z mechanisms, 42%). In the same period, the EDL^15^ microphone has recorded a total of 56 min of Martian sounds, mainly during rover operations (for example, rover drive, arm motion). Extended Data Table 1 lists all acoustic files used or mentioned in this study, except for those shown in Figs. 3a, 4a, which are too numerous to cite individually (see below).

SuperCam’s microphone records air pressure fluctuations from 20 Hz to 12.5 kHz at a 25-kHz sampling frequency, and up to 50 kHz when the 100-kHz sampling mode is used. The analogue signal from the microphone, ranging from 0 to 5 V, is digitized (12-bit depth) using one of four electronic gains to boost the sensitivity from 0.6 to 21 V Pa^−1^ and the resolution from 2 to 0.06 mPa. Gain 0 is used to record the accompanying sounds from LIBS sparks on calibration targets, gains 1, 2 and 3 to record the accompanying sounds from LIBS sparks at various distances and gain 3 is used for atmospheric recordings. Recordings of the atmosphere and mechanical noises are generally 167 s long. The EDL microphone can record 10 mn and longer time series, with a fixed gain amplifier followed by a 24-bit/44-kHz digitizer (the key characteristics are summarized in Extended Data Table 2).

Typical LIBS sequences consist of 30 laser shots at the same position fired at 3 Hz (for technical reasons, only 29 shots are recorded). For a given target, such a sequence is usually repeated 5–10 times, on fresh sampling points separated by a few millimetres. The laser-induced acoustic signal is monitored at a 100-kHz sampling frequency for 60 ms around each laser pulse. The start of the recording window is precisely timed on the laser trigger, so that the propagation time of the sound wave can be measured with an uncertainty <10 μs. Up to Sol 216, SuperCam’s microphone has recorded sound sequences for 123 Martian targets located at distances ranging from 2.05 m (target Garde) to 8.01 m (target Pepin) from the microphone. On seven occasions, it has also recorded the acoustic signal related to LIBS measurements of the titanium (Ti) calibration target^44^ located on the rover deck 1.51 m from the microphone.

For the derivation of the sound speed (Fig. 3a), targets farther than 6 m are excluded because of a small signal-to-noise ratio that prevents a good time-of-flight measurement. Recordings of the LIBS acoustic signals from Ti are also excluded, as their sound propagates above the rover and are biased by extra heating and turbulence induced by the warm body of the rover. In total, 109 targets between Sol 1 and Sol 216 are considered for Fig. 3. For the attenuation study (Fig. 4), regolith or loose material targets, which generally lead to a lower sound amplitude, are excluded, as well as out-of-focus points for the same reason. In total, 96 targets are used. The measurements from Ti are included and provide a useful constraint on the attenuation at short distance. As the laser energy used on Ti is lower than the laser energy used on Mars targets (110 A pumping current on Ti compared with 155 A on Mars targets), the PSD amplitude from the Ti measurements are normalized by a factor of 155/110, as the pumping current is proportional to the laser energy, which is proportional to the laser irradiance, as the spot size and the pulse duration remains the same.

### Dataset of artificial sounds

Both EDL and SuperCam microphones are also used to record sounds produced by the rover. They help to inform operators about equipment health (for example, rover driving, MOXIE) and provide sources of sound that are well localized in space and time (for example, during Ingenuity flights or LIBS sparks). All recordings also pick up some perturbations, such as intense single-frequency emissions at 195 Hz, 198.75 Hz and the following harmonics at 780 Hz and 795 Hz that result from the rover’s internal heat pump used for thermal management. Sounds of the rover’s instruments or pumps propagate through both structural vibrations (microphonics) and acoustic propagation in the atmosphere.

**Extended Data Fig. 1:**
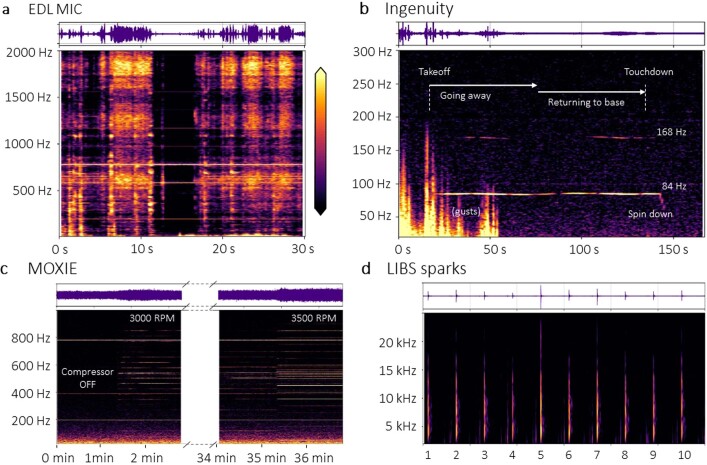
Artificial sounds recorded by Perseverance. For each panel: time is along the *x* axis; on top time series data in mPa (see hereafter for the range when it exists); at the bottom PSD in Pa^2^ Hz^−1^ (a colour scale is shown next to panel **a**; ranges are different for each panel but not indicated). **a**, Unfiltered spectrogram of a segment (240–270 s) of the 16-min EDL microphone recording during the rover drive (Sol 16). Time series data are not calibrated in pressure. **b**, Spectrogram of Ingenuity’s fourth flight (Sol 69). Time series data range from −10 mPa to +10 mPa. **c**, Unfiltered spectrogram of MOXIE oxygen production (Sol 81). Time series data range from −2.5 mPa to +2.5 mPa. **d**, Series of ten LIBS shots on target Hedgehog (Sol 37), labelled 1 to 10. Time series data range from −500 mPa to 500 mPa.

The EDL microphone^15^ was used to record the rover drive on Sol 16 (Extended Data Fig. 1a). Broad, quasi-continuous ‘screech’ signals in the 520–700 Hz, 1.2–1.4 kHz and 1.6–1.9 kHz bandwidths are assumed to arise directly from frictional interaction of the metal wheel tread with surface rocks. Sonorous transients or ‘clanks’ are seen at 13 s with several narrow-frequency components but with a lower total sound intensity than the aforementioned phenomenon. It is suggested that these are structural resonances of mobility system elements (for example, suspension) excited by near-impulsive changes in loading, for example, when a wheel slips off the edge of a rock.

The Ingenuity rotorcraft^33^ provides a localized but moving source of sound on Mars. On Sol 69 during the fourth flight, SuperCam’s microphone recorded the entire 116-s duration of the flight. A prominent acoustic signal, up to 2 × 10^−7^ Pa^2^ Hz^−1^ (1.5 mPa sound pressure level), associated with the BPF at 84 Hz and its first overtone 168 Hz was detected by SuperCam’s microphone (Extended Data Fig. 1b). All phases of the flight are visible but the take-off occurred during a gust (at the rover location) as high as 20 mPa. The BPF clearly stands out but its overtone is much fainter, owing to greater atmospheric absorption at higher frequency. After landing, the microphone captured the blades spinning down.

The MOXIE instrument^43^ operates every 1–2 months to produce a few grams of gaseous O_2_. The primary objective of these repeated operations is to look for possible degradation of the O_2_ production efficiency associated with the harsh environment of Mars. MOXIE uses SuperCam’s microphone recordings for independent diagnosis of compressor performance, including precise measurements of the motor rotation rates, as indicated by the fundamental frequency of the observed comb of harmonics. Distinct transitions in Extended Data Fig. 1c, recorded during a night-time run (Sol 81), correspond to commanded changes in motor speed from 50 to 58.3 Hz. The loudest harmonics are near 500 Hz, at which several more frequencies are also excited. This range corresponds to resonant frequencies of the MOXIE instrument, as observed during dynamics testing. Even recorded during one of the quietest times of the day, the amplitude of the signal only reaches 1.5 mPa.

Recording LIBS sparks was the main rationale to develop SuperCam’s microphone to infer physical properties of rock targets, such as their hardness^13^. Typical LIBS sequences consist of 30 laser shots at the same position per observation, fired at 3 Hz (Extended Data Fig. 1d). LIBS operations are monitored by the microphone at a 100-kHz sampling rate for 60 ms around each laser pulse. The mean amplitude of the signal is 0.25 ± 0.08 Pa (1σ) for this shot sequence.

### Wind speed, temperature and pressure data

The wind, temperature and pressure data are recorded from the MEDA^21^ instrument. The wind data are acquired up to 2 Hz, pressure at 1 Hz and temperature at 1 or 2 Hz. Wind speed and direction are independently acquired from two individual booms separated by 120° (termed boom 1 and boom 2), for which one is preferred for a given wind direction (see accuracies and resolutions in Extended Data Table 2).

The MCD^32^ provides climate predictions derived from 3D simulations of Mars atmosphere performed with the Mars global climate model developed at the Laboratoire de Météorologie Dynamique (http://www-mars.lmd.jussieu.fr). The Laboratoire de Météorologie Dynamique Mars global climate model is described in ref. ^32^ but — since then — it has adopted more sophisticated and realistic modelling for the CO_2_, dust and water cycles, photochemistry, radiative transfer and so on. In this work, we use the climatology scenario^45^ from the MCD Version 5.3, in which: (1) the simulated spatial and vertical dust distributions are reconstructed from observations during Martian years 24 to 31 without global dust storms (thus representative of standard climate conditions) and (2) average solar extreme ultraviolet conditions are assumed. In this study, the MCD outputs (surface temperature and atmospheric temperature at 2 m above the surface) are provided for daytime local times in increments of 1 h and between Ls = 5.2° and Ls = 104.7° in increments of 10° to capture the seasonal variations in temperature.

### Spectral analysis of acoustic data

The microphone data from SuperCam are converted from volts to pascals using the instrument sensitivity for each gain (0.6, 1.3, 5.3 and 21.6 V Pa^−1^, corresponding to amplification factors of 29 to 972). The microphone’s electronic response function for each gain (bandpass filter between 100 Hz and 10 kHz) is used to correct raw spectra below 100 Hz and above 10 kHz. EDL microphone data are not converted into physical units. PSDs represented in Fig. 1, 2b were computed from a Fourier transform, using a Welch’s estimator. Spectrograms represented in Fig. 2a and Extended Data Fig. 1b, c are computed with a Hanning window of 2 s. Extended Data Figure 1a is computed with a window of 1 s and Extended Data Fig. 1d with a window of 5 ms.

### Time series (laser-induced spark recordings)

**Extended Data Fig. 2:**
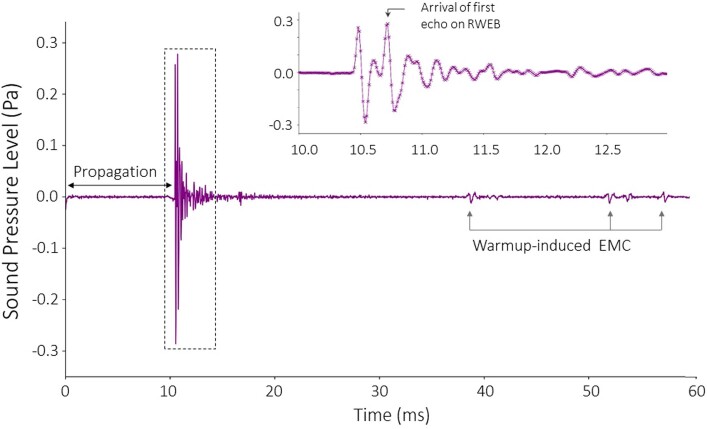
Recording of laser-induced shock wave. Target Hedgehog (point #1, shot #1). The inset focuses on the time when the acoustic wave is recorded.

The creation of the laser-induced plasma is accompanied by a shock wave, which can be described as an N-wave acoustic pulse^46^ primarily, a short, approximately 300-μs-long compression/rarefaction acoustic signal. This signal is followed by echoes on nearby rocks and the rover structure, plus diffraction. The whole acoustic signal typically lasts less than 5 ms. A bandpass filter is applied to remove electromagnetic interferences, atmospheric signal below 2 kHz and to reduce noises above 20 kHz. There are residuals of the laser warm-ups but they do not affect the determination of the sound speed (Extended Data Fig. 2).

Time series data are used to calculate the local speed of sound. The distance to each target, which is returned by the instrument’s autofocus, is known to an accuracy of ±0.5% (ref. ^8^). The laser trigger time is known to a few microseconds and the shock wave becomes sonic after 1 μs (ref. ^12^), which is less than 0.1% of the propagation time. The arrival of the pressure wave is considered to be detected when the signal increases 3σ above the background.

### Doppler effect (Ingenuity recording)

**Extended Data Fig. 3:**
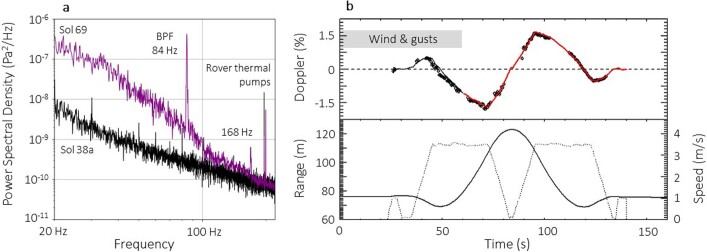
Recording of the BPF of Ingenuity’s fourth flight. **a**, PSD for the entire recording (purple). The rover’s thermal pumps are shown at 195 Hz and 198.75 Hz. PSD for Sol 38a is the reference of a quiet day. **b**, Top, variations of the received frequency along the trajectory of the flight (diamond symbol). During the first 60 s, the recording is perturbed by high winds. In red, the Doppler effect is modelled with *f* = 84.44 Hz at the source and *c* = 237.7 m s^−1^. Bottom, range between the rover and Ingenuity (solid line, left *y* axis) and rotorcraft speed (dotted line, right *y* axis).

The fourth (Sol 69), fifth (Sol 76), sixth (Sol 91) and eighth (Sol 120) flights of Ingenuity were recorded by SuperCam at a 25-kHz sampling rate. We use data from the fourth flight, as this flight came closer to Perseverance than any other flight SuperCam could record. During this flight, Ingenuity climbed to an altitude of 5 m, accelerated to 3.5 m s^−1^, travelled 130 m at constant height, decelerated, turned around and returned to its base by the same route. Taking off at a distance of 76 m from Perseverance, it came as close as 69 m and moved as far as 123 m from Perseverance (Extended Data Fig. 3b, bottom).

On the PSD obtained during the whole recording, the BPF (two times the rotation rate for a two-bladed rotor) at 84 Hz and its first harmonic at 168 Hz are clearly visible above the background, which itself is higher than that of Sol 38a, a very quiet recording on Mars (Extended Data Fig. 3a). There is no other tone above the background. There is a period of atmospheric turbulence up to 56 s into the recording that explains why the spectrum at low frequency is above that of Sol 38a. Discontinuities in the amplitude of the tones at 84 Hz and 168 Hz are visible during the cruise phase of the flight. Such a modulation beat results from the interferences of two signals with slightly different frequencies (about 50 mHz apart), each originating from the two blades that are frequency shifted. The study of this phase shift is outside the scope of this paper.

Each tone is fitted by a Gaussian function every 0.5 s. In the main text, we report on the study of the BPF at 84 Hz. The received frequency varies along Ingenuity’s flight (Extended Data Fig. 3b, top) as a function of the variation of the distance range between the rover and the helicopter. The received frequency, the classical Doppler effect, varies by ±1.5%. The fit of this tone, when the atmosphere is quiet (*t* > 60 s), as a function of the range rate, yields *f* = 84.44 Hz for the BPF at the source and *c* = 237.7 ± 3 m s^−1^. A similar fit to the first harmonic yields *f* = 168.90 Hz and *c* = 236.9 ± 4 m s^−1^, which are coherent with values derived from the BPF.

### Sound attenuation with distance

As the sound wave propagates through an atmosphere, part of the acoustic energy is transferred to the propagation medium as heat by an absorption mechanism called atmospheric (or intrinsic) attenuation. This process has been largely described and validated for the atmosphere on Earth^34,47,48^. The atmospheric attenuation, linked to the motion of molecules, depends on the frequency of the wave. It can be attributed to two phenomena. First, the classical attenuation includes the heat loss caused by viscous friction and losses from non-adiabatic heat diffusion between the compression and rarefaction areas. This phenomenon is all the more important for short-period waves, when there is less time to establish an equilibrium. The classical attenuation is proportional to the square of the frequency. The second phenomenon is the molecular attenuation, owing to the excitation of the internal degrees of freedom of the polyatomic molecules (rotational and vibrational modes), each one taking some time, called ‘relaxation time’, to return to equilibrium. The shorter the period of the wave, compared with the relaxation time, the less time molecules have to relax their energy and hence the greater the absorption of the acoustic energy.

This theory has been applied to the atmosphere of Mars to compute empirical attenuation models^3,4^ (Fig. 4). Although Mars models are in good agreement above 10 kHz (classical attenuation is well constrained thanks to a good knowledge of dynamic viscosity and thermal conductivity for CO_2_ as a function of temperature), they differ strongly in the infrasonic and part of the audible range, depending on the way molecular relaxation is modelled. The Williams model^4^ extrapolates experimental data for CO_2_ that have been acquired at 1 bar and above 273 K. The model considers that molecular attenuation increases proportionally to the frequency, reaches a maximum value at the relaxation frequency, *f*_R_ (240 Hz), and then decreases as 1/*f*. On the other hand, the Bass and Chambers model^3^ discriminates molecular relaxation into rotational and vibrational relaxations. Rotational relaxation is modelled by *f* squared, just as in classical attenuation. For vibrational relaxation, the model considers that, below *f*_R_, vibrational attenuation grows as *f* squared. Above *f*_R_, vibration modes are not excited and the vibrational attenuation stays at a constant level.

As a supplementary note, CO_2_ has three vibrational modes at 1,341 cm^−1^ (*ν*_1_ symmetric stretching), 667 cm^−1^ (*ν*_2_ degenerate bending) and 2,349 cm^−1^ (*ν*_3_ asymmetric stretching). Associated vibrational temperatures are 1,890 K, 960 K and 3,360 K, respectively. At 240 K, the respective contribution of each mode to the vibrational specific heat are 3.7% (*ν*_1_), 96.3% (*ν*_2_) and <0.1% (*ν*_3_) at 240 K. This justifies why first-order models can only consider the contribution of the *ν*_2_ bending mode to the vibrational specific heat.

## Online content

Any methods, additional references, Nature Research reporting summaries, source data, extended data, supplementary information, acknowledgements, peer review information; details of author contributions and competing interests; and statements of data and code availability are available at 10.1038/s41586-022-04679-0.

## Figures and Tables

**Extended Data Table 1:**
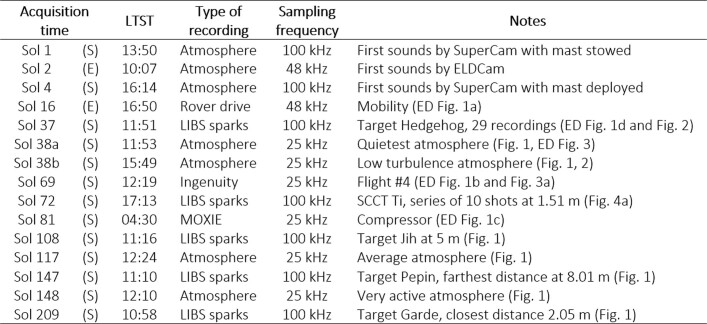
List of audio recordings used in this study

Recordings from the microphones of SuperCam (S) and EDL (E), with the Local True Solar Time (LTST) of each acquisition.

**Extended Data Table 2:**
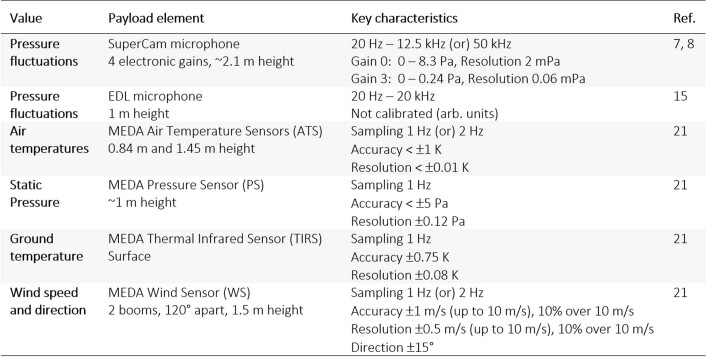
Origin and characteristics of the dataset used in this study

Audio recordings are provided by the microphones of SuperCam and EDL. Atmospheric data are provided by MEDA. Resolution and accuracy are defined as the 90% confidence range of the statistical error of the pressure, temperature or wind speed values^8,21^.

## Data Availability

All acoustic data are publicly available at the Planetary Data System Geosciences Node: 10.17189/1522646.
