# Author Correction: In situ recording of Mars soundscape

**DOI:** 10.1038/s41586-022-05050-z

**Published:** 2022-07-21

**Authors:** S. Maurice, B. Chide, N. Murdoch, R. D. Lorenz, D. Mimoun, R. C. Wiens, A. Stott, X. Jacob, T. Bertrand, F. Montmessin, N. L. Lanza, C. Alvarez-Llamas, S. M. Angel, M. Aung, J. Balaram, O. Beyssac, A. Cousin, G. Delory, O. Forni, T. Fouchet, O. Gasnault, H. Grip, M. Hecht, J. Hoffman, J. Laserna, J. Lasue, J. Maki, J. McClean, P.-Y. Meslin, S. Le Mouélic, A. Munguira, C. E. Newman, J. A. Rodríguez Manfredi, J. Moros, A. Ollila, P. Pilleri, S. Schröder, M. de la Torre Juárez, T. Tzanetos, K. M. Stack, K. Farley, K. Williford, the SuperCam team, R. C. Wiens, T. Acosta-Maeda, R. B. Anderson, D. M. Applin, G. Arana, M. Bassas-Portus, R. Beal, P. Beck, K. Benzerara, S. Bernard, P. Bernardi, T. Bosak, B. Bousquet, A. Brown, A. Cadu, P. Caïs, K. Castro, E. Clavé, S. M. Clegg, E. Cloutis, S. Connell, A. Debus, E. Dehouck, D. Delapp, C. Donny, A. Dorresoundiram, G. Dromart, B. Dubois, C. Fabre, A. Fau, W. Fischer, R. Francis, J. Frydenvang, T. Gabriel, E. Gibbons, I. Gontijo, J. R. Johnson, H. Kalucha, E. Kelly, E. W. Knutsen, G. Lacombe, S. Le Mouélic, C. Legett, R. Leveille, E. Lewin, G. Lopez-Reyes, E. Lorigny, J. M. Madariaga, M. Madsen, S. Madsen, L. Mandon, N. Mangold, M. Mann, J.-A. Manrique, J. Martinez-Frias, L. E. Mayhew, T. McConnochie, S. M. McLennan, N. Melikechi, F. Meunier, G. Montagnac, V. Mousset, T. Nelson, R. T. Newell, Y. Parot, C. Pilorget, P. Pinet, G. Pont, F. Poulet, C. Quantin-Nataf, B. Quertier, W. Rapin, A. Reyes-Newell, S. Robinson, L. Rochas, C. Royer, F. Rull, V. Sautter, S. Sharma, V. Shridar, A. Sournac, M. Toplis, I. Torre-Fdez, N. Turenne, A. Udry, M. Veneranda, D. Venhaus, D. Vogt, P. Willis

**Affiliations:** 1 Institut de Recherche en Astrophysique et Planétologie, Université de Toulouse 3 Paul Sabatier, CNRS, CNES, Toulouse, France; 2 Space and Planetary Exploration Team, Los Alamos National Laboratory, Los Alamos, NM USA; 3 Institut Supérieur de l’Aéronautique et de l’Espace (ISAE-SUPAERO), Université de Toulouse, Toulouse, France; 4 Space Exploration Sector, Johns Hopkins Applied Physics Laboratory, Laurel, MD USA; 5 Present Address: Department of Earth, Atmospheric, and Planetary Sciences, Purdue University, West Lafayette, IN USA; 6 Institut de Mécanique des Fluides, Université de Toulouse 3 Paul Sabatier, INP, CNRS, Toulouse, France; 7 Laboratoire d’Etudes Spatiales et d’Instrumentation en Astrophysique, Observatoire de Paris, CNRS, Sorbonne Université, Université Paris Diderot, Meudon, France; 8 Laboratoire Atmosphères, Milieux, Observations Spatiales, CNRS, Université Saint-Quentin-en-Yvelines, Sorbonne Université, Guyancourt, France; 9 Universidad de Málaga, Málaga, Spain; 10 Department of Chemistry and Biochemistry, University of South Carolina, Columbia, SC USA; 11 Jet Propulsion Laboratory, California Institute of Technology, Pasadena, CA USA; 12 Institut de Minéralogie, de Physique des Matériaux et de Cosmochimie, CNRS, Sorbonne Université, MNHN, Paris, France; 13 Heliospace Corporation, Berkeley, CA USA; 14 Haystack Observatory, Massachusetts Institute of Technology, Westford, MA USA; 15 Department of Aeronautics and Astronautics, Massachusetts Institute of Technology, Cambridge, MA USA; 16 Laboratoire de Planétologie et Géosciences, CNRS, Nantes Université, Université Angers, Nantes, France; 17 Escuela de Ingeniería de Bilbao, Universidad del País Vasco UPV/EHU, Bilbao, Spain; 18 Aeolis Corporation, Sierra Madre, CA USA; 19 Centro de Astrobiología (INTA-CSIC), Madrid, Spain; 20 Deutsches Zentrum für Luft- und Raumfahrt (DLR), Institute of Optical Sensor Systems, Berlin, Germany; 21 Blue Marble Space Institute of Science, Seattle, WA USA; 22 University of Hawai‘i at Mānoa, Mānoa, HI USA; 23 U.S. Geological Survey, Flagstaff, AZ USA; 24 University of Winnipeg, Winnipeg, Canada; 25 University of the Basque Country UPV/EHU, Leioa, Bilbao Spain; 26 Institut de Planétologie et Astrophysique de Grenoble, CNRS, Université Grenoble Alpes, Grenoble, France; 27 Department of Earth, Atmospheric and Planetary Sciences, Massachusetts Institute of Technology, Cambridge, MA USA; 28 Centre Lasers Intenses et Applications, CNRS, CEA, Université de Bordeaux, Bordeaux, France; 29 Plancius Research, Severna Park, MD USA; 30 Laboratoire d’Astrophysique de Bordeaux, CNRS, Université de Bordeaux, Bordeaux, France; 31 Centre National d’Études Spatiales, Toulouse, France; 32 Université de Lyon, UCBL, ENSL, UJM, CNRS, LGL-TPE, Villeurbanne, France; 33 Groupe d’Instrumentation Scientifique, Observatoire Midi-Pyrénées, Toulouse, France; 34 GeoRessources, CNRS, Université de Lorraine, Nancy, France; 35 California Institute of Technology, Pasadena, CA USA; 36 University of Copenhagen, Copenhagen, Denmark; 37 McGill University, Montreal, Canada; 38 University of Valladolid, Valladolid, Spain; 39 Agencia Estatal Consejo Superior de Investigaciones Científicas, Madrid, Spain; 40 Department of Geological Sciences, University of Colorado Boulder, Boulder, CO USA; 41 University of Maryland, College Park, MD USA; 42 State University of New York, Stony Brook, NY USA; 43 Department of Physics and Applied Physics, Kennedy College of Sciences, University of Massachusetts Lowell, Lowell, MA USA; 44 Institut d’Astrophysique Spatiale, CNRS, Université Paris-Saclay, Orsay, France; 45 Institut Universitaire de France, Paris, France; 46 University of Nevada, Las Vegas, Las Vegas, NV USA

**Keywords:** Atmospheric dynamics, Atmospheric dynamics, Characterization and analytical techniques

Correction to: *Nature* 10.1038/s41586-022-04679-0 Published online 1 April 2022

In the version of this article initially published, the Acknowledgements statement was missing the text “Part of this research was carried out at the Jet Propulsion Laboratory, California Institute of Technology, under a contract with the National Aeronautics and Space Administration (80NM0018D0004),” which has now been inserted in the HTML and PDF versions of the article.

